# Recovery, overloading, and protein interactions in asymmetrical flow field-flow fractionation

**DOI:** 10.1007/s00216-019-01673-w

**Published:** 2019-02-21

**Authors:** Maria Marioli, Wim Th. Kok

**Affiliations:** Analytical Chemistry Group, van’t Hoff Institute for Molecular Sciences, University of Amsterdam, Postbus 94157, 1090 GD Amsterdam, The Netherlands

**Keywords:** Field-flow fractionation, Protein interactions, Aggregates, Ultrafiltration membranes, Recovery, Overloading

## Abstract

In asymmetrical flow field-flow fractionation (AF4), similar to other separation techniques, mass recovery and overloading require special attention in order to obtain quantitative results. We conducted a systematic study with five globular proteins of different molecular weight (36.7–669 kDa) and isoelectric point (4.0–6.5), and ultrafiltration membranes that are commonly used in aqueous AF4, regenerated cellulose (RC) and polyethersulfone (PES). Phosphate-buffered saline (PBS) with ionic strength 0.15 M and pH 7.2 was used as the carrier liquid in this study. The actual molecular weight cutoff (MWCO) was found to be higher than the nominal value and varied between membranes of different chemistry but the same nominal MWCO. Adsorption on the membrane was found to be dependent on the membrane chemistry (RC had lower adsorption compared to PES), and independent of the protein standard for the examined proteins. On the other hand, the mass overloading effects (i.e., higher retention times, peak broadening, and fronting peaks) were significantly more pronounced for γ-globulin than for the other proteins. The overloading effects could be rationalized with the increase of the local viscosity close to the membrane, depending on the properties of the proteins, and we derived theoretical equations that related the dependency of the migration velocity on the protein concentration through this non-ideal viscosity effect.

**Electronic supplementary material:**

The online version of this article (10.1007/s00216-019-01673-w) contains supplementary material, which is available to authorized users.

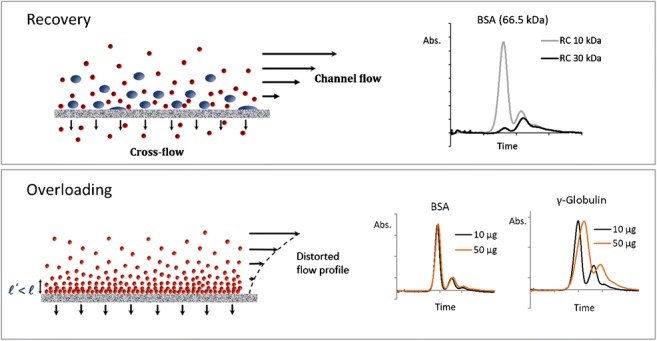

## Introduction

Devised by Giddings half a century ago [1], field-flow fractionation (FFF) has emerged as a powerful tool for the characterization of macromolecules and nanoparticles [2–4]. The asymmetrical variant of flow FFF [5], abbreviated as AsFlFFF or AF4, is a mild size-based separation technique which has found various applications in biopharmaceuticals [6, 7]. It has been demonstrated that flow FFF is able to give higher recoveries and better resolution for the large aggregates of antibodies compared to size exclusion chromatography (SEC) [8–10]. SEC is conventionally applied for the quantification of the protein aggregates, but these may be filtered out by the column, elute unresolved in the void volume, or adsorb on the chromatographic support due to non-specific interactions, especially the large ones [8, 11]. Furthermore, the sample dilution inside the AF4 channel is lower than that in the SEC column, decreasing the chances of dissociation of reversible aggregates [10].

**Fig. 1:**
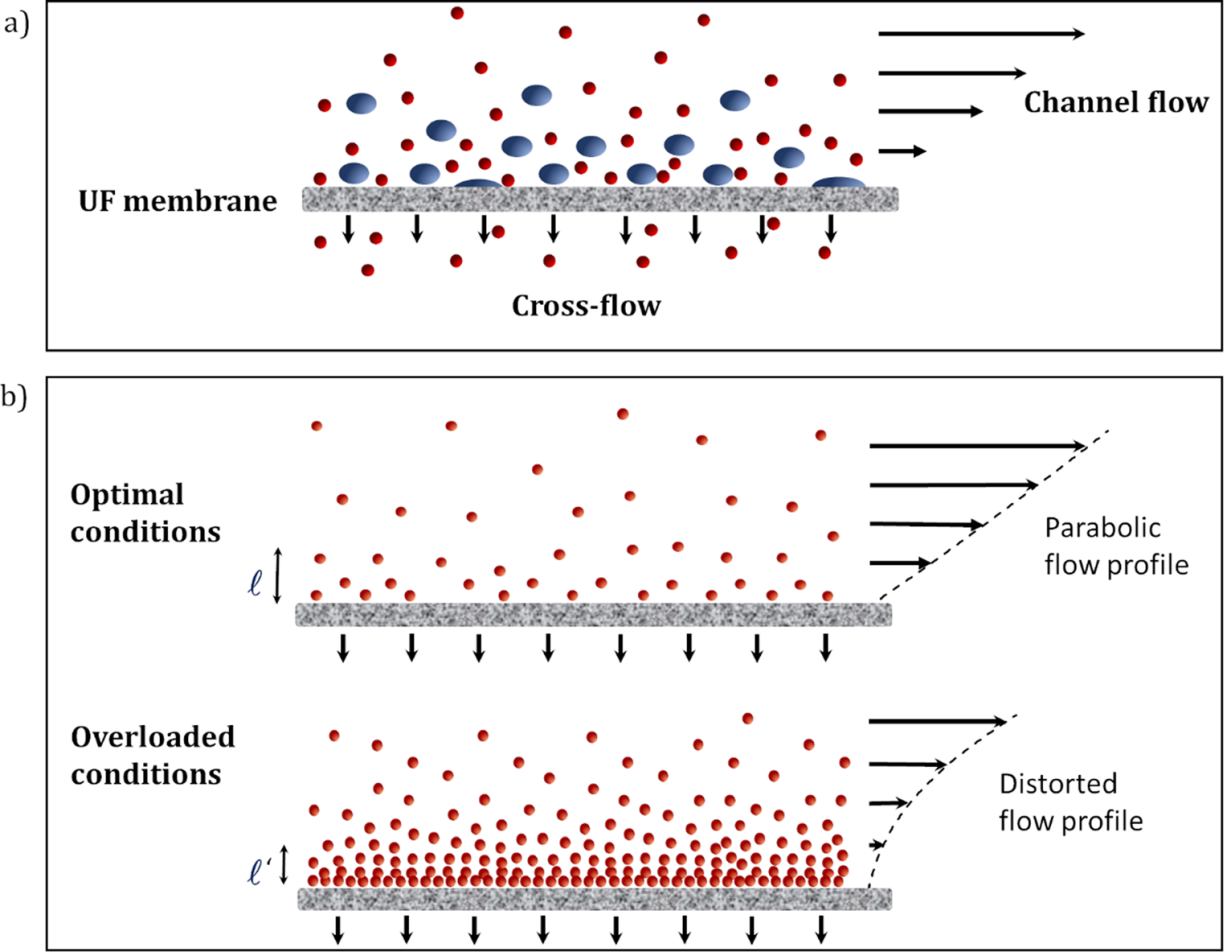
**a** Low recovery may have several causes such as protein permeability or protein adsorption on the ultrafiltration (UF) membrane. **b** High sample load may decrease the mean distance from the membrane because of the concentration-dependent diffusivity and may alter the parabolic profile because of the concentration-dependent viscosity. The size of the proteins is exaggerated for visual purposes

However, to obtain accurate results with AF4, a careful method optimization is required with respect to recovery, resolution, sensitivity, and reproducibility [12–19]. High recoveries can be achieved (> 90%) for proteins with a proper method optimization but they rarely reach 100% [9, 15, 20–22] as there are several factors that may contribute to sample loss (Fig. 1a):

- The proteins may penetrate the pores if their size is smaller, or clog them, if their size is comparable to the pore size, which may lead to fouling.
- The proteins may interact with the membrane as a result of chemical interaction (adsorption) or physical interaction (surface roughness).
- Part of the sample may elute in the void peak, if the focusing is not sufficient, or when the cross-flow stops, if the cross-flow program is not optimized to elute large aggregates.
- Sample is retained and lost at the edges of the channel spacer (edge effects).
- Protein-protein interactions may occur if the carrier liquid is not a good solvent.

In addition, there might be sample loss for reasons not related to the AF4 method (e.g., sample not well solubilized, protein degradation due to sample preparation and storage, protein adsorption on the labware/injection system/tubing). Moreover, the recovery might appear lower due to incorrect determination (e.g., related to the limit of detection of concentration detectors).

High cross-flow rates improve resolution but may decrease recovery as they force the proteins closer to the membrane (smaller mean layer thickness *ℓ*) for more time, increasing the probability of interaction. For this reason, it has been suggested that recovery could be considered dependent on the mean layer thickness, and/or on the ratio of the mean channel flow velocity to the cross-flow velocity [15, 17]. In addition, the composition of the carrier liquid is crucial [15, 16]; protein–membrane interactions (e.g., electrostatic, van der Waals, hydrophobic) may lead to protein adsorption on the membrane, particularly in low ionic strength solutions [16]. Furthermore, proteins become less soluble at very low or very high ionic strength (salting in–salting out phenomena) and at a pH close to their isoelectric point.

High sample load improves detection when AF4 is used as an analytical technique, and throughput when it is used as a semi-preparative technique, but it may cause overloading. For proteins and polymers, the retention times typically increase and peaks become fronting [12, 23]. The opposite effect, a decrease in retention time and tailing peaks, is observed for very large macromolecules because of steric repulsion or shear-induced elongation, and for charged macromolecules in low ionic strength solvents, because of strong electrostatic repulsion [14, 24, 25]. Caldwell et al. attributed the overloading effects for macromolecules to the concentration-dependent viscosity that distorts the axial flow profile, and to the concentration-dependent diffusivity that changes the mean layer thickness [23], as depicted in Fig. 1b.

Various studies have been conducted to investigate the factors that lead to sample loss and mass overloading. However, few have investigated a larger selection of proteins and membranes [15, 21] or attempted to explain the overloading theoretically [23, 26–28]. We conducted a systematic study comparing five proteins of different molecular weight (MW) and isoelectric point (pI) and different membranes, and we derived a theoretical equation that predicts the reduction in zone velocity because of the viscosity effect.

## Theory

Previous theoretical studies have solved the differential equations numerically for specific solutes [23, 26] or analytically [27, 28] to explain overloading. The ones that have derived analytical solutions have taken into account the mean distance effect and ignored the viscosity effect [27] or considered only the contribution from the intrinsic viscosity [28]. However, at the concentrations that develop on the membrane under overloaded conditions, the local viscosity depends not only on the intrinsic viscosity but also on the intermolecular interactions. Here we derive equations considering the viscosity increase due to pairwise interactions.

First, it is assumed that the concentration of the protein as a function of the distance from the wall *x* can be described in the usual way,

1 $$ c(x)={c}_0\bullet \exp \left(-\frac{x}{\ell}\right) $$

where *c*_0_ is the protein concentration at the wall and ℓ is the equilibrium layer thickness which, for the useful retention levels, is equal to the mean layer thickness and equal to

2 $$ \ell =\frac{D}{u_{cr}} $$

where *D* is the diffusion coefficient of the protein and *u*_*cr*_ is the cross-flow velocity on the membrane. It is further assumed that the shear stress in the protein layer is constant and equal to the shear stress in the linear part of the parabolic profile of the unperturbed channel flow *τ*_0_, so that the local flow velocity *v*(*x*) can be found from

3 $$ \frac{dv(x)}{dx}=\frac{\tau_0}{\eta (c)} $$

where *x* is the distance from the membrane and *η*(*c*) is the concentration-dependent viscosity of the carrier solution. Equation (3) is expected to be valid for a wide range of concentrations since it has been demonstrated that despite the high viscosity of concentrated protein solutions, the solution remains a Newtonian fluid even for concentrations > 400 mg/mL [29].

The solution viscosity *η* increases with the protein concentration *c*, relative to the solvent viscosity *η*_0_, as a virial polynomial expansion [30],

4 $$ \eta (c)={\eta}_0\left(1+{k}_1c+{k}_2{c}^2+\cdots \right) $$

where *k*_1_ is the intrinsic viscosity [*η*], which is the contribution of the single molecules, and the higher order terms depend on the intermolecular interactions. When the cubic and higher order terms are omitted, Eq. (4) is known as the Huggins equation.

**Fig. 2:**
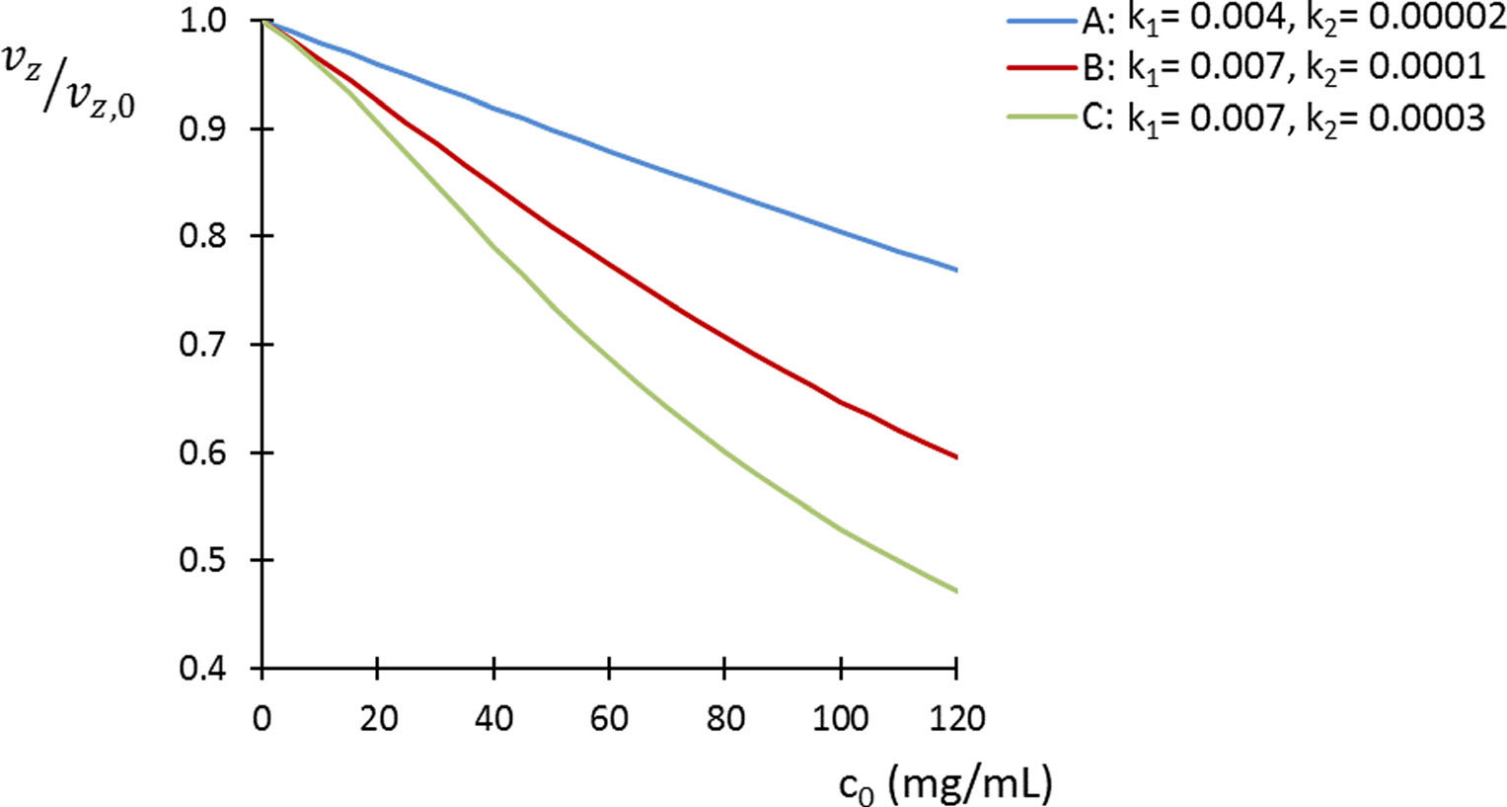
Reduction of the zone velocity due to the viscosity effect (Eq. (5)) with respect to the concentration at the wall for different type of proteins; *k*_1_ (mL/mg) and *k*_2_ (mL^2^/mg^2^) were derived from the literature from the intrinsic viscosity and the Huggins constant A for BSA [34], and B and C for monoclonal antibodies [35]

By substitution of Eqs. (1) and (4) in Eq. (3), and integration over *x*, the velocity profile can be found. The zone velocity can then be found by integration of the product of the concentration and the velocity profiles. The result for the zone velocity *v*_*z*_ is then

5 $$ {v}_z=\left\{\begin{array}{c}{v}_{z,0}\bullet \frac{2}{c_0\bullet \sqrt{4{k}_2-{k}_1^2}}\bullet \arctan \left(\frac{c_0\bullet \sqrt{4{k}_2-{k}_1^2}}{2+{k}_1{c}_0}\right),\kern0.5em 4{k}_2>{k}_1^2\\ {}{v}_{z,0}\bullet \frac{2}{c_0\bullet \sqrt{-4{k}_2+{k}_1^2}}\bullet \operatorname{arctanh}\left(\frac{c_0\bullet \sqrt{-4{k}_2+{k}_1^2}}{2+{k}_1{c}_0}\right),\kern0.5em 4{k}_2<{k}_1^2\end{array}\right. $$

where *v*_*z*, 0_ is the velocity of the zone under ideal conditions (*c*_0_ → 0). In Fig. 2, the predicted effect of the sample load on the zone velocity is shown for proteins of different *k*_1_ and *k*_2_ values; when the viscosity increase is more in a quadratic way (*k*_2_*c* > *k*_1_), there is a more sudden change of the velocity above a certain threshold mass load. Assuming a Gaussian concentration profile along the channel, the maximum concentration (Gaussian center) for well-retained solutes is [31],

6 $$ {C}_{00}(z)=\frac{m_{inj}}{b(z)\cdotp \ell \cdotp \sqrt{2\pi \cdotp {\sigma}_l^2(z)}} $$

where *m*_*inj*_ is the injected mass, *b*(*z*) is the channel breadth, and the peak variance in units of length ${\sigma}_l^2(z)$ can be estimated from the plate height.

The mean distance effect, originating from the dependency of diffusivity on concentration, has been neglected so far. The diffusivity may also be expressed as a polynomial expansion of the concentration which includes the intermolecular interactions and the viscosity effects [32],

7 $$ D={D}_0\left(1+{k}_D\bullet \mathrm{c}+\bullet \bullet \bullet \right) $$

where *k*_*D*_ is the interaction parameter, and *D*_0_ is the diffusion coefficient at infinite dilution. When intermolecular interactions are predominantly attractive, then *k*_*D*_ ≤ 0, and the mean distance will decrease shifting the curve in Fig. 2 towards lower values. Higher sample viscosity entails lower (more negative) *k*_*D*_ since both are related to strong intermolecular interactions [33] making the differences in zone velocity between proteins more pronounced.

The theory (Eq. (5)) demonstrates that the increase in retention time at high injected mass does not depend only on the sample concentration at the membrane (which depends on the operational parameters and channel dimensions according to Eq. (6)) but also on the sample properties which affect the viscosity of concentrated solutions. For a more detailed description of the theory, see the Electronic Supplementary Material (ESM).

## Methods and materials

### Instrumentation

The AF4 system was an Eclipse DualTec system (Wyatt Technology Europe, Dernbach, Germany) which was connected to an Agilent HPLC system 1200 (Agilent Technologies, Waldbronn, Germany) that consisted of a degasser, an isocratic pump, an inline PVDF filter 0.1 μm (Millipore, MA, USA), a UV detector, and an autosampler equipped with a thermostat and an injection loop of 100 μL. SEC experiments were performed with a BioSEC 300 Å, 5 μm, 4.6 × 300 mm column (Agilent Technologies). The AF4 channel had a tip-to-tip length of 17.3 cm. The spacer had a nominal thickness of 350 μm, and a trapezoidal shape with a maximum breadth of 2.15 cm and a minimum of 0.3 cm. The ends were tapered with lengths 2.0 cm and 0.3 cm from the inlet and outlet respectively. The total area of the accumulation wall was 20.5 cm^2^. Nadir PES membranes (MWCO 5 kDa and 10 kDa) and Milllipore RC membranes (MWCO 10 kDa and 30 kDa) precut for the AF4 channel (Superon, Dernbach, Germany) were used.

A mathematical computing software (Mathematica) was used to solve the integrals mentioned in the Theory section.

### Samples and carrier solution

Five standard proteins, i.e., β-lactoglobulin (variants A and B) from bovine milk, bovine serum albumin (BSA), γ-globulin from bovine serum (Cohn fraction II), apoferritin from equine spleen, and thyroglobulin from bovine thyroid, were purchased from Sigma-Aldrich (Sigma-Aldrich, St. Louis, MO, USA). All protein standards were in lyophilized form, except apoferritin which was in solution, at a concentration of 37 mg/mL. The purity of the protein content was > 97% for all the standards. PBS 0.15 M (20 mM due to sodium phosphate salts) and pH = 7.2 was used as a carrier liquid for both SEC and AF4 experiments and as a diluent for the proteins. Protein solutions were prepared by dissolving or diluting protein standards to a concentration of 1 mg/mL which were placed in the autosampler without filtration. The protein solutions and the PBS buffer were prepared daily before experiments.

### SEC and AF4 methods

UV detection was at 280 nm unless stated otherwise, and the temperature of the autosampler was set at 5 °C to prevent protein degradation or aggregation during experiments. For SEC experiments, the injection volume was 10 μL and the flow rate was 0.3 mL/min. For the AF4 experiments, the sample was focused for 3 min with a focus flow of 1.5 mL/min and the focusing point was set at 16% of the channel length, which corresponded to a focusing position of 2.7 cm from the channel inlet. The injection inlet was permanently closed with a flat bottom plug and during focusing the sample was injected from the inlet. The elution of the sample was performed in the constant cross-flow mode. Pretreatment with saturation of 50 μg BSA was performed for every new membrane once before experiments to enhance recovery. The injected mass for the recovery experiments and the measurements of the diffusion coefficients was 10 μg (10 μL injection volume). For the overloading experiments, the injected mass was 10 μg and 50 μg unless stated otherwise.

## Results and discussion

### Measurement of hydrodynamic radii

The diffusion coefficients and the hydrodynamic (Stokes) radii were estimated for all the proteins. First, BSA was used as calibrant with known diffusion coefficient, equal to 6.21·10^−11^ m^2^/s [36], to estimate the channel thickness as it was proposed by Litzén [13]. The actual channel thickness was estimated to be 260 μm with a RC 10 kDa membrane, cross-flow rate ${\dot{V}}_c$ = 1 mL/min, and outlet flow rate ${\dot{V}}_{\mathrm{out}}$ = 1 mL/min, significantly reduced compared to the nominal value because of membrane compressibility. Then, the diffusion coefficients for the other proteins were estimated under the same conditions from their retention times and their hydrodynamic radii by the Stokes–Einstein equation. The experimental results are given in Table 1 together with additional information from the literature. Dimers had hydrodynamic radii ~ 1.35 times larger than the monomers. In addition, the actual channel thickness with a PES 10 kDa membrane using same method was estimated as 297 μm. Similar differences in compressibility between these membranes have been reported previously [37].

**Table 1:** Physicochemical properties of the standard proteins used in this study

Protein	MW (kDa)	D (m^2^ s^−1^) ^b^	*R*_H_ (nm) ^c^	pI
β-Lactoglobulin	36.7 ^a^	8.04·10^−11^	2.8	5.1, 5.3 [38]^d^
BSA	66.5	6.21·10^−11^ [36]	3.6	4.7 [36]
γ-Globulin	150	4.22·10^−11^	5.3	6.5 (average) [39]
Apoferritin	443	3.31·10^−11^	6.8	4.0 [40]
Thyroglobulin	669	2.43·10^−11^	9.2	4.6 [41]

^a^The MW of the β-lactoglobulin monomer is 18.35 kDa but it is present as a dimer in neutral pH [42]

^b^Diffusion coefficients were estimated experimentally using BSA as calibrant

^c^Hydrodynamic radii were estimated from the Stokes–Einstein equation

^d^The pI of β-lactoglobulin is 5.1 and 5.3 for the variants A and B respectively [38]

### Recovery experiments

**Fig. 3:**
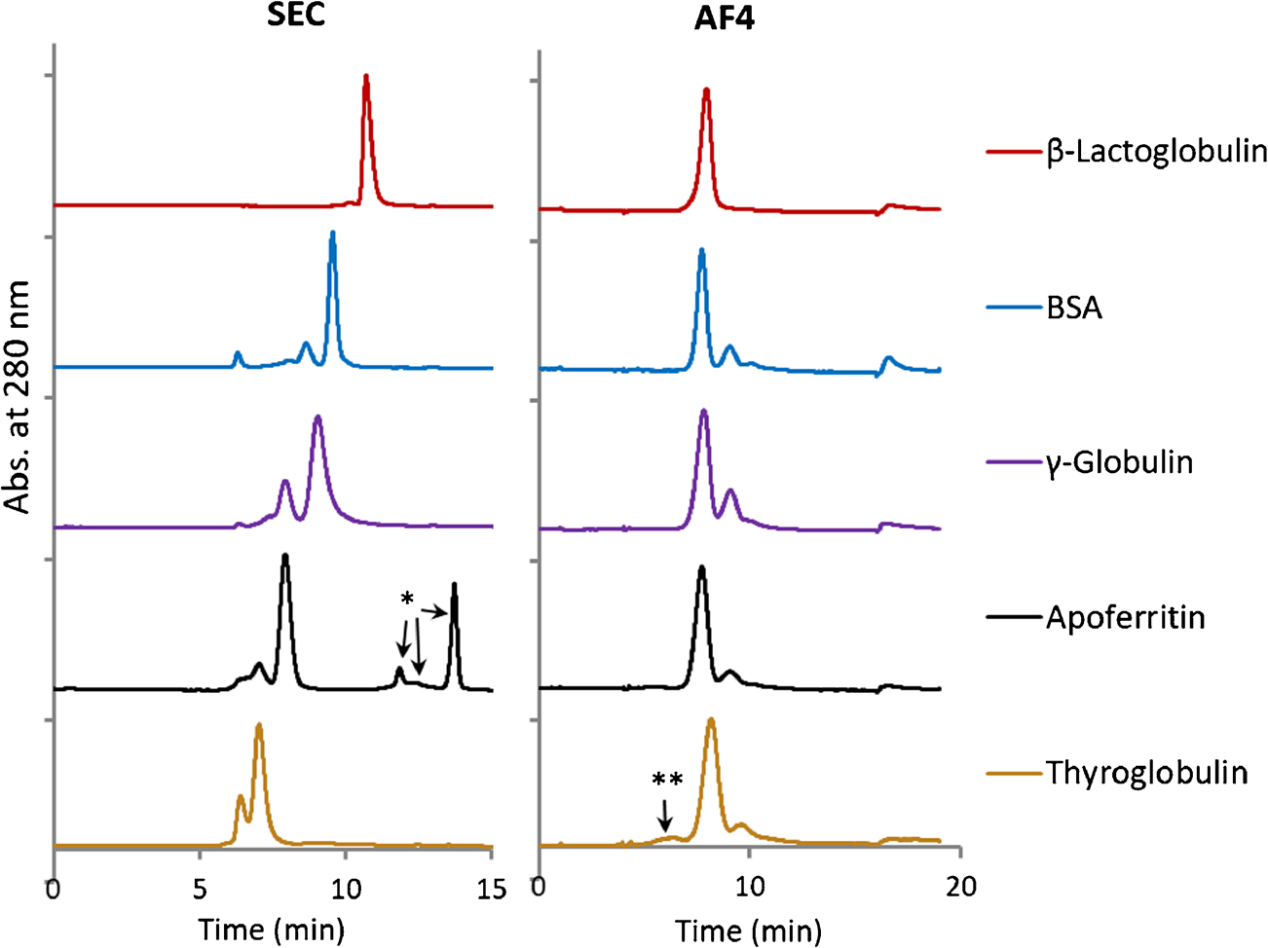
SEC chromatographs and AF4 fractograms of the standard proteins. SEC conditions: 0.30 mL/min flow rate, AF4 conditions: RC 10 kDa membrane; elution starts at *t* = 4 min, ${\dot{V}}_c$ and ${\dot{V}}_{\mathrm{out}}$ vary for each protein, see Table 2

Recoveries were estimated from the ratio of the peak area (area under the curve) of the fractionated sample to the peak area of the unfractionated sample, measured by injecting the sample through the channel at the same outlet flow without applying focus or cross-flow. This method assumes that the unfractionated sample does not contain low MW components that absorb at the same wavelength. Therefore, additional SEC experiments were carried out which revealed that apoferritin contained a fraction of low MW components (marked as * in Fig. 3) that absorbed at 280 nm. The fraction of the response at 280 nm that corresponded to apoferritin was estimated to be ~ 67% of the total sum of peak areas, and to account for this, the recovery of apoferritin was corrected by this factor in the AF4 experiments.

Recovery was calculated only from peak areas that corresponded to protein monomers and oligomers (dimers, trimers, etc.). Higher order aggregates that could elute when the cross-flow stopped were not included in the recovery. Thyroglobulin contained a smaller MW component (marked as ** in Fig. 3) that eluted before the monomer which was included in the integration.

First, the recoveries of the three smaller proteins (β-lactoglobulin, BSA, and γ-globulin) were compared at different cross-flow rates (1–3 mL/min) and constant outlet flow rate. The retention time was ~ 30% lower with PES membranes than with RC membranes for the same flow rates because of the difference in the actual channel thickness. To make sure that any measured difference in recovery between the membranes was not due to the higher retention times, the outlet flow rates were adjusted experimentally, at 0.8 mL/min and 1.2 mL/min for RC and PES membranes, respectively, which resulted to similar retention times (within 5%). Run-to-run duplicate analysis was performed and the average RSD for the peak area was ~ 2%. In addition, the membrane-to-membrane variation was estimated in duplicate using a new membrane of the same type and nominal MWCO.

**Fig. 4:**
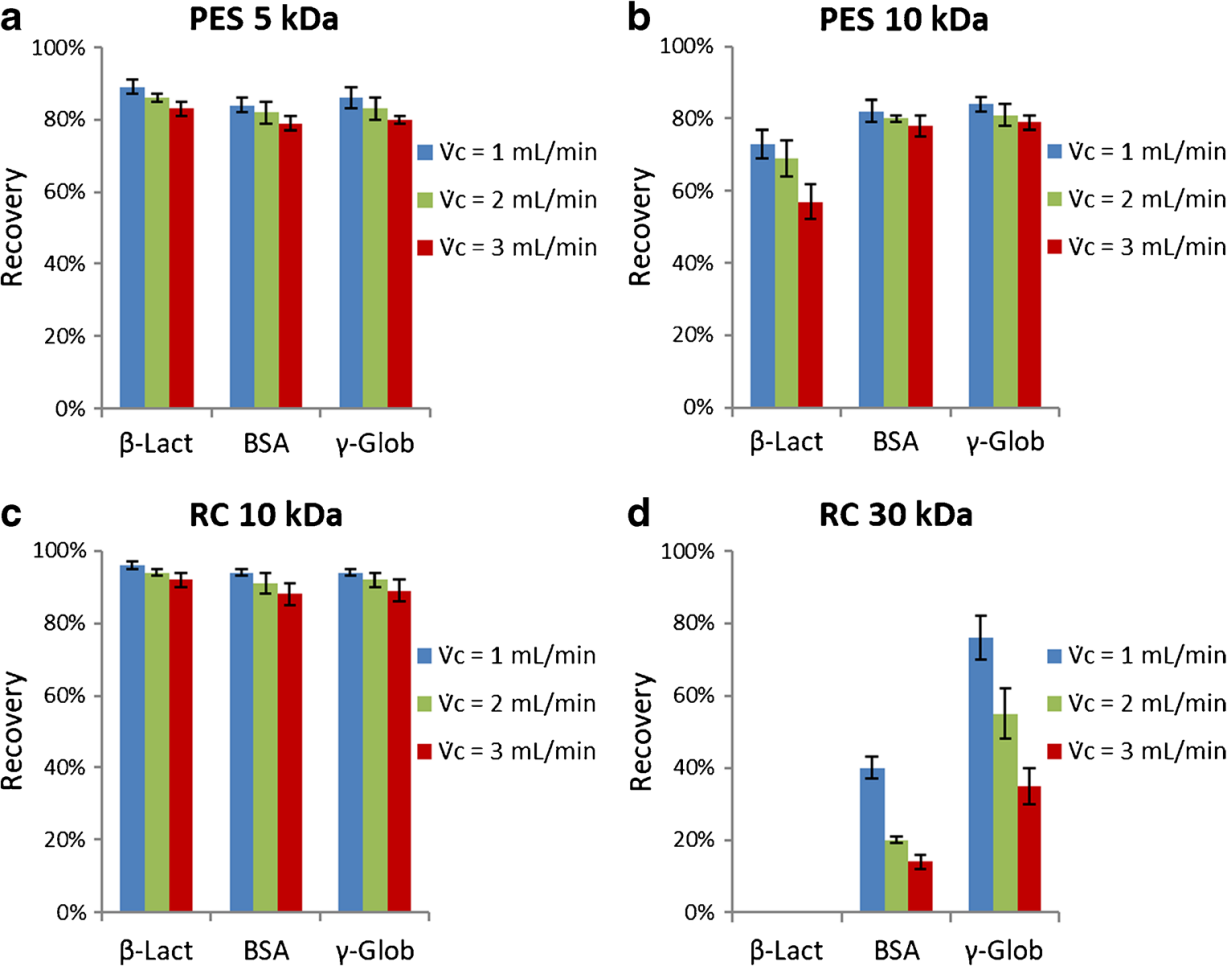
**a**–**d** Protein recovery of β-lactoglobulin (36.7 kDa), BSA (66.5 kDa), and γ-globulin (150 kDa) estimated with different UF membranes and cross-flow rates; ${\dot{V}}_{\mathrm{out}}$ = 0.8 mL/min for RC and ${\dot{V}}_{\mathrm{out}}$ = 1.2 mL/min for PES membranes. The error bars represent the membrane-to-membrane variation and are given at 1σ level (± σ)

**Fig. 5:**
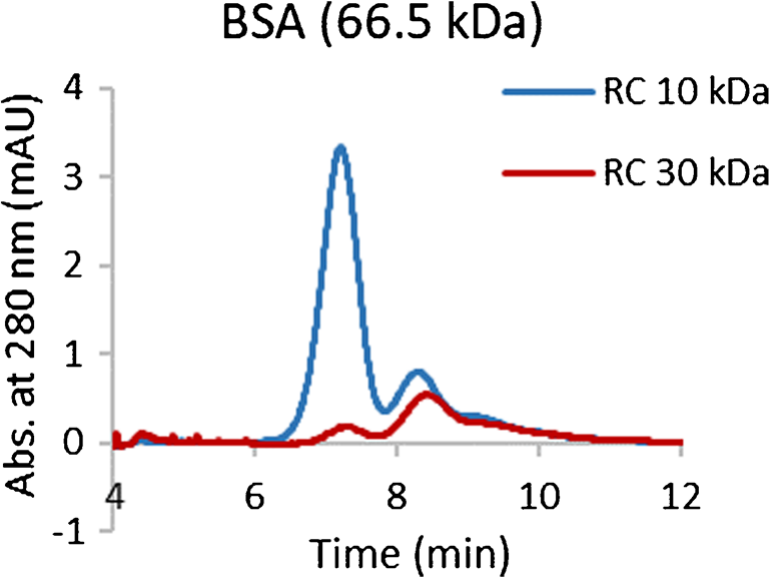
Fractograms of BSA with RC 10 and 30 kDa, ${\dot{V}}_c$ = 2.0 mL/min and ${\dot{V}}_{\mathrm{out}}$ = 0.8 mL/min

The results, displayed in Fig. 4, reveal that the actual MWCO is higher than the nominal value for both RC and PES membranes, since the differences in protein recovery between membranes of the same chemistry and different nominal MWCO could be attributed to the MWCO values. In particular, the actual MWCO of the PES 10 kDa was found to be higher since β-lactoglobulin (36.7 kDa) had a lower recovery with this membrane (Fig. 4b) than with PES 5 kDa (Fig. 4a). Similarly, the actual MWCO was found to be higher for the RC 30 kDa (Fig. 4d) since β-lactoglobulin (36.7 kDa), BSA (66.5 kDa), and γ-globulin (150 kDa) had lower recoveries with this membrane compared to RC 10 kDa (Fig. 4c). The BSA fraction that was recovered with the RC 30 kDa corresponded mainly to oligomers (Fig. 5) which highlights the importance of obtaining high recovery values to ensure a high proportionate recovery.

The fact that the actual MWCO was found higher than the nominal value is not surprising as there are no industrial standards to determine the nominal value [43], which is usually defined as the MW of the smallest macromolecule that exhibits more than 90% rejection. The type of the macromolecule (dextran, PEG, protein), the solvent, the operational conditions, and the device (stirred cell or tangential flow system) have an impact in the determination of the MWCO [43–45]. For globular proteins, it is suggested to use a membrane with a nominal MWCO several times lower than their molecular weight when polysaccharides have been used for the determination of the nominal MWCO, since proteins are more compact than polysaccharides [46]. Next to that, AF4 operates in a very different manner than the methods/devices that are typically used for the MWCO determination (e.g., with very low amounts of proteins).

PES 10 kDa (Fig. 4b) and RC 10 kDa (Fig. 4c) have the same nominal MWCO but exhibited different rejection of β-lactoglobulin. As it was mentioned above, the manufacturers might have used different methods (macromolecules, device, operational conditions, etc.) to determine the nominal MWCO. Differences in protein permeability for membranes of different chemistry and the same nominal MWCO have been reported previously, where a RC 30 kDa membrane had smaller actual MWCO and narrower pore distribution (4.4–4.7 nm) compared to a polysulfone 30 kDa membrane (4.1–5.7 nm) [43].

As anticipated, regardless of the membrane and protein sample, higher cross-flow rates resulted in lower recoveries, and the decrease was more noticeable when protein permeability was the main cause of sample loss (Fig. 4). Higher cross-flow rates force the solutes closer to the membrane (smaller *ℓ*) and for a longer time, increasing the possibility for them to penetrate the pores or to adsorb on the membrane surface. Kassalainen and Williams [15] suggested that the dependency of the recovery on the cross-flow rate could be described as related to the mean layer thickness or to the ratio of the average channel flow velocity to the cross-flow velocity 〈*v*〉/*u*_*cr*_ which affects the zone velocity. Wahlund suggested that the latter seems more reasonable since good recoveries have been reported at very low *ℓ*-values (*ℓ* ≈ 1 μm) and high flow conditions [17].

To investigate if there is sample loss related to causes other than membrane permeability, RC 10 kDa and PES 5 kDa membranes were chosen for further investigation as they showed high protein rejection even for the smaller protein (β-lactoglobulin). For the same flow rates, thyroglobulin has ~ 3 times lower *ℓ*-value and ~ 3 times longer retention time compared to β-lactoglobulin, and consequently slightly higher probability to interact with the membrane. Therefore, to compare the protein standards, cross-flow and outlet flow rates are adjusted to achieve the same *ℓ*-value and retention time *t*_R_ for each protein. First, the required cross-flow rates ${\dot{V}}_c$ were estimated from Eq. (2) for *ℓ* = 3 μm and the diffusion coefficients in Table 1. Secondly, the required outlet flow rates ${\dot{V}}_{\mathrm{out}}$ were estimated theoretically (with a small adjustment if needed), for *t*_*R*_ = 4 min, according to the equation for well-retained compounds,

8 $$ {t}_R=\frac{w^2}{6D}\ln \left(1+\frac{{\dot{V}}_c}{{\dot{V}}_{\mathrm{out}}}B\right) $$

where *B* is the fraction of the channel area over which protein elution occurs.

The results are tabulated in Table 2; the exact theoretical mean layer thickness was 2.9 ± 0.1 μm and the experimental retention time was 4.0 ± 0.2 min. RC 10 kDa showed recoveries of ~ 90% and PES 5 kDa of ~ 80% for all proteins, while the recoveries were similar for monomers and oligomers with both membranes (high proportionate recovery). Overall, the results did not indicate a correlation of the recovery with the protein standard; thyroglobulin had slightly but not significantly lower values. Li et al. have found higher recoveries (95–98%) with a RC 10 kDa membrane for BSA, γ-globulin, and thyroglobulin, determined with a frit inlet channel [21]. By using a frit inlet, a mild hydrodynamic relaxation occurs instead of the focusing process, which may result in higher recoveries.

**Table 2:** Protein recovery for mean layer thickness ℓ = 3 μm and retention time *t*_R_ = 4 min

Protein	Flow rates (mL/min)	ℓ (μm)	*t*_R_ (min) ± s.d.	Recovery (%) ± s.d.
Regenerated cellulose (RC) 10 kDa				
β-Lactoglobulin	${\dot{V}}_c$ = 3.5, ${\dot{V}}_{\mathrm{out}}$ = 0.6	2.9	4.03 ± 0.10	91 ± 2
BSA	${\dot{V}}_c$ = 2.7, ${\dot{V}}_{\mathrm{out}}$ = 0.8	2.9	3.80 ± 0.07	88 ± 3
γ-Globulin	${\dot{V}}_c$ = 1.8, ${\dot{V}}_{\mathrm{out}}$ = 1.0	2.9	3.91 ± 0.09	92 ± 2
Apoferritin	${\dot{V}}_c$ = 1.4, ${\dot{V}}_{\mathrm{out}}$ = 1.1	3.0	3.86 ± 0.11	90 ± 2
Thyroglobulin	${\dot{V}}_c$ = 1.1, ${\dot{V}}_{\mathrm{out}}$ = 1.3	2.8	4.02 ± 0.22	84 ± 4
Polyethersulfone (PES) 5 kDa				
β-Lactoglobulin	${\dot{V}}_c$ = 3.5, ${\dot{V}}_{\mathrm{out}}$ = 0.9	2.9	4.04 ± 0.30	82 ± 2
BSA	${\dot{V}}_c$ = 2.7, ${\dot{V}}_{\mathrm{out}}$ = 1.2	2.9	4.08 ± 0.13	79 ± 3
γ-Globulin	${\dot{V}}_c$ = 1.8, ${\dot{V}}_{\mathrm{out}}$ = 1.4	2.9	4.22 ± 0.09	84 ± 3
Apoferritin	${\dot{V}}_c$ = 1.4, ${\dot{V}}_{\mathrm{out}}$ = 1.5	3.0	4.11 ± 0.02	82 ± 4
Thyroglobulin	${\dot{V}}_c$ = 1.1, ${\dot{V}}_{\mathrm{out}}$ = 1.7	2.8	4.21 ± 0.03	75 ± 3

The recovery was similar for all the protein standards, but it should be noted that the proteins were analyzed in a carrier liquid of physiological ionic strength (PBS 0.15 M) and at a pH (7.2) higher than the pI values of the proteins (Table 1) and of the membranes [47, 48]. Previous studies showed that at low ionic strength conditions (which favor electrostatic interactions) significant adsorption may occur when the solutes have a charge opposite to that of the membrane [49] or when the pH is close to their isoelectric point [14]. Kassalainen and Williams measured much lower recoveries for γ-globulin compared to BSA at similar pH as this study but at low ionic strength, presumably because the pI of γ-globulin is closer to the pH of the buffer [15]. In addition, all protein standards used in this study are water-soluble globular proteins commonly used as calibration standards in SEC. Proteins with very high surface hydrophobicity or larger structural flexibility could have higher adsorption on the membranes, and therefore much lower recoveries, at the same experimental conditions.

PES 5 kDa exhibited ~ 10% lower recoveries compared to RC 10 kDa (Table 2) which could be attributed to protein adsorption on the membrane. The same conclusion can be drawn comparing the recoveries obtained with PES 10 kDa (Fig. 4b) and RC 10 kDa (Fig. 4c), for the proteins that exhibited high rejection (BSA and γ-globulin). These findings are consistent with those of previous studies that showed higher adsorption of biomolecules on PES membranes in AF4 [15, 50]. Salinas-Rodriguez et al. [47] and Alele et al. [48] compared the properties of ultrafiltration RC and PES membranes and concluded that both are hydrophilic (contact angle < 90°) but that RC is more hydrophilic than PES. PES membranes have a high degree of hydrophobicity because of the aromatic groups but they are often chemically or physically modified in order to become more hydrophilic [51].

The fact that the recoveries did not reach 100% might be a result of several causes other than hydrophobic adsorption on the membrane as suggested by our experiments. First, the method was not designed to include the higher order aggregates, something that it is typically performed adding a step with a cross-flow gradient program. Secondly, edge effects might have also contributed to sample loss since the solutes could be trapped and lost in the edges. However, the channel aspect ratio is typically very high and therefore the sample amount that reaches the edges is very small. Lastly, surface roughness could also have reduced recovery as molecules can be trapped inside cavities.

### Overloading experiments

**Fig. 6:**
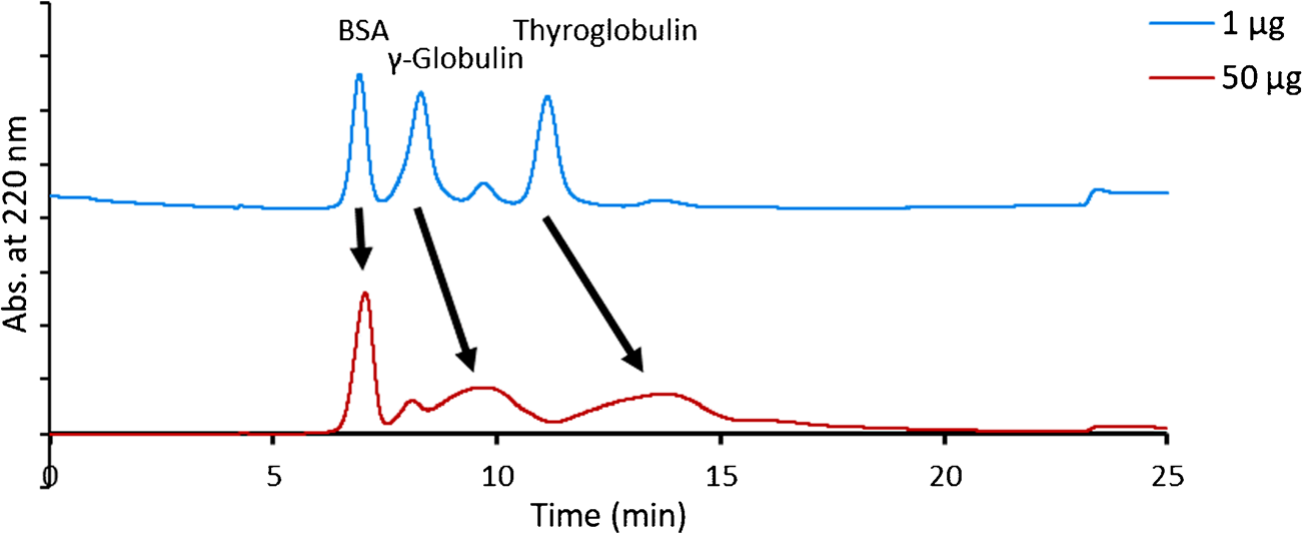
Overloading effect. The peaks are normalized for visual comparison; AF4 conditions: ${\dot{V}}_c$ = 3.0 mL/min, ${\dot{V}}_{\mathrm{out}}$ = 1.5 mL/min, RC 10 kDa

High sample load of a protein mixture (BSA, γ-globulin, thyroglobulin) resulted in a shift of the retention times and band broadening, where the more retained components were more affected (Fig. 6). This is not surprising since for the same flow rates, larger proteins are accumulating closer to the membrane resulting in higher concentrations. Assuming that the nonequilibrium effect is the largest contribution in plate height [31] and the mean axial velocity is constant along the channel, it can be estimated from Eq. (6) that the maximum concentration *C*_00_(*z*) is proportional to *D*^−2^. Consequently, each protein in Fig. 6 has much higher (~ 2 times) *C*_00_(*z*) than the less retained component.

To investigate if the properties of the proteins influence loading, we need to compare them with similar concentration on the accumulation wall. Like in the recovery experiments, the flow rates were adjusted to obtain *ℓ* = 3 μm and *t*_*R*_ = 4 min, for the membranes that exhibited high rejection of all the proteins (RC 10 kDa and PES 5 kDa). Under these conditions, the theoretical plate height is slightly different for each protein because of the difference in the flow rates, which results in slightly but not significantly lower *C*_00_(*z*) for the proteins of higher MW. The effects of overloading were assessed with the increase in plate height between the optimal (10 μg) and high (50 μg) sample load conditions. The plate height was calculated from

9 $$ H=\left(L-{z}^{\prime}\right)\frac{\sigma_t^2}{t_R^2} $$

where *σ*_*t*_ is the standard deviation in time units derived from the peak width at half height, *L* is the channel length, and *z*^′^ is the focusing point. From the experimental plate height of the proteins in Table 3 and Eq. (6), it can be estimated that *C*_00_(*z*) was 14–21 mg/mL for all the proteins in the middle of the migration path for 10-μg injected mass. Therefore, it could reach concentrations 70–105 mg/mL for 50-μg injected mass, and even higher close to the focusing point.

**Table 3:** Retention time and plate height under low (10 μg) and high (50 μg) injected sample mass, for mean layer thickness *ℓ* = 3 μm and retention time *t*_*R*_ = 4 min. The average RSD for the retention time is 3% and for the plate height is 4%

	*t*_*R*_ (min)		H (mm)		Increase in H (mm) ± s.d.
Protein	10 μg	50 μg	10 μg	50 μg	
Regenerated cellulose (RC) 10 kDa					
β-Lactoglobulin	4.03	4.29	0.49	0.86	0.37 ± 0.03
BSA	3.80	3.89	0.46	0.55	0.08 ± 0.01
γ-Globulin	3.91	4.44	0.77	1.71	0.92 ± 0.02
Apoferritin	3.86	3.92	0.76	0.89	0.12 ± 0.01
Thyroglobulin	4.02	4.14	0.97	1.20	0.21 ± 0.08
Polyethersulfone (PES) 5 kDa					
β-Lactoglobulin	4.04	4.30	0.51	0.75	0.24 ± 0.03
BSA	4.08	4.23	0.48	0.63	0.16 ± 0.02
γ-Globulin	4.22	5.08	0.87	1.70	0.83 ± 0.02
Apoferritin	4.11	4.33	0.91	0.99	0.08 ± 0.06
Thyroglobulin	4.21	4.59	1.10	1.30	0.21 ± 0.01

**Fig. 7:**
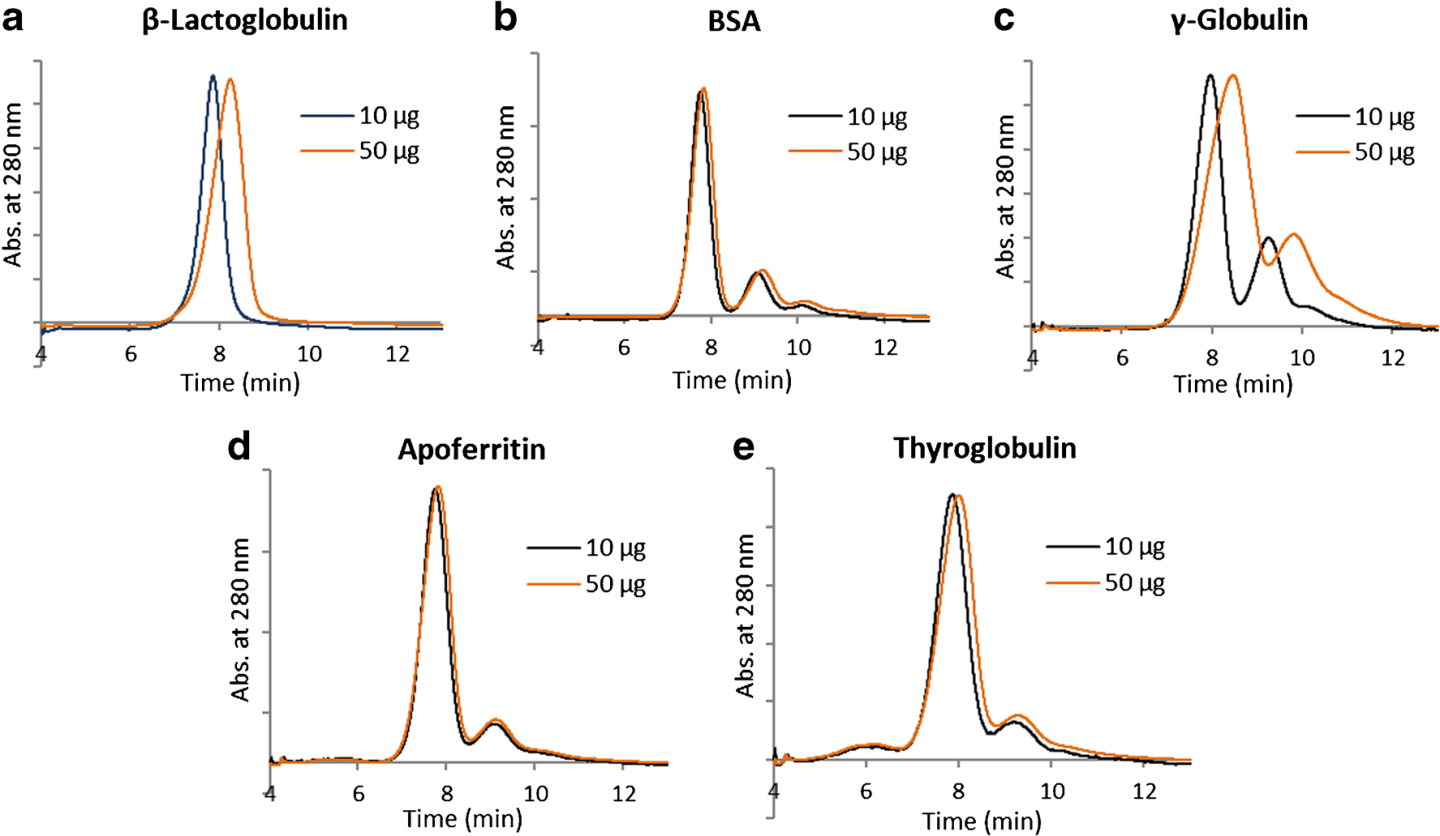
Overloading effect in AF4. For the same mean layer thickness (ℓ = 3 μm) and retention time (*t*_*R*_ = 4 min), γ-globulin exhibits significantly higher increase in retention time and in plate height followed by β-lactoglobulin. Peaks are scaled for visual comparison. Experimental conditions: RC 10 kDa membrane, elution starts at *t* = 4 min, ${\dot{V}}_c$ and ${\dot{V}}_{\mathrm{out}}$ vary for each protein (see Table 2)

All protein standards exhibited an increase in retention time and in plate height although at a considerable different degree (Table 3, Fig. 7). Regardless of the membrane, γ-globulin had the highest increase in plate height followed by β-lactoglobulin. BSA and apoferritin showed only marginal changes, which might be related to a small increase in diffusivity with concentration due to electrostatic repulsion that could partly cancel out the viscosity effect. For β-lactoglobulin (Fig. 7a) and γ-globulin (Fig. 7c), for which the overloading effects were more pronounced, we observe that the overloaded fractograms exhibit fronting peaks with the same peak onset, which may be attributed to the viscosity effect. In agreement with our conclusions, viscosity measurements of concentrated γ-globulin and BSA solutions have shown significantly higher viscosities for the former in PBS [52]. Specifically, solutions of 150 mg/mL concentration resulted in viscosities of approximately 2 and 4 mPa s for BSA and γ-globulin respectively.

The more pronounced overloading effects observed for γ-globulin could be explained with a higher intrinsic viscosity (higher *k*_1_ in Eq. (4)) or stronger intermolecular attraction (higher *k*_2_ in Eq. (4)). The average pI of γ-globulin (Table 1) is closer to the pH of the buffer (pH = 7.2) which may induce less repulsion, giving rise to attractive interactions. Moreover, the γ-globulin standard used in this study (Cohn Fraction II), is a mixture of immunoglobulins with a very broad range of pI values 5.2–9.2 [53], which means that at neutral pH oppositely charged species may exist that attract each other.

However, β-lactoglobulin showed also noticeable overloading effects, while the pI is much lower than the pH of the buffer. This indicates that protein interactions are not only dependent on the pI values. In diluted solutions, long-range (electrostatic) interactions are significant, but in concentrated solutions, mid- or short-range interactions (van der Waals, hydrophobic, excluded volume, etc.) become as important [54]. It has been found that β-lactoglobulin increases the viscosity in whey protein concentrates to a larger extent compared to other components [55]. In addition, it has a high dipole moment and unusual salt-in effects in high NaCl concentrations, which has been attributed to a unique charge distribution on the surface [56]. An asymmetric surface charge density may cause strong attraction between positively charged patches on the surface of the protein and negatively charged regions. Yadav et al. investigated the factors that lead to viscous solutions of monoclonal antibodies and concluded that the effective volume, the net charge, and the charge distribution are crucial [35].

The viscosity increase is a result of self-association because of the intermolecular interactions, which has been described as a transient network of molecules [35]. In AF4, this self-association does not seem to result in irreversible aggregation, since the relative concentration of the γ-globulin oligomers is similar (~ 30%) under normal and overloaded conditions (Fig. 7c). Previous studies of concentrated protein solutions have shown that the concentration of the irreversible oligomers increased only in concentrations > 250 mg/mL [57]. Moreover, irreversible oligomers are formed by non-native proteins such as partly unfolded forms, which requires a lag time [58] and the AF4 analysis including focusing is typically kept very short (< 20 min).

Membrane adsorption could also be an effect of overloading. However, this would likely lead to tailing peaks and/or lower recoveries. The recovery difference between optimal and overloaded conditions was similar for all the proteins and membranes within 4%. Nevertheless, the injected mass was only moderately increased; previous studies have demonstrated a reduction in recovery with very high sample loads [22].

The results demonstrate that not only the injected mass and the operational conditions are crucial, but also the type of the protein and presumably the solvent. Previous studies have investigated wider channels to increase sample loading [59]; we suggest that changes in the carrier liquid that aim to reduce the increase in viscosity with increasing sample concentration could also significantly increase the loading capacity. However, protein interactions are very complex, and therefore the factors that decrease viscosity are case specific. It has been reported that salt addition reduced the viscosity for one type of monoclonal antibody and increased it for another type [60]. A temperature increase could also increase loading, since at high temperatures the viscosity is less dependent on the concentration (as long as the temperature remains below the high temperature limit that thermal denaturation occurs) [61].

## Conclusions

In this study, the recovery and overloading of five globular proteins (MW = 36.7–669 kDa, pI = 4.0–6.5) were evaluated with different membranes and PBS with ionic strength 0.15 M and pH 7.2 as the carrier liquid. For the smaller proteins, protein permeability was the main cause of sample loss since the actual MWCO of the membranes was higher than the nominal value, and different for membranes of different chemistry but the same nominal MWCO (i.e., PES 10 kDa and RC 10 kDa). For the membranes that exhibited low protein permeability, PES 5 kDa and RC 10 kDa, the recovery was ~ 80% and ~ 90% respectively for all the proteins, when determined at the same mean layer thickness and retention time.

Although the results indicate that protein-membrane adsorption depends on the membrane material (RC exhibited lower protein adsorption compared to PES) and not on the protein standard, it is important to note that the experiments were carried out at physiological ionic strength and at a pH where all proteins and membranes were negatively charged. Previous studies using carrier liquids with low ionic strength have shown differences in recovery between protein standards, presumably because of differences in their pI values (low ionic strength solutions favor electrostatic interactions) or differences in their structural stability [14–16]. Furthermore, the results of this study should not be extended to every protein type since proteins with very hydrophobic surface or more flexible structures could adsorb much stronger on the membrane at the same experimental conditions.

Sample loading was dependent not only on the operational conditions but also on the protein standard; γ-globulin showed considerably more pronounced overloading effects. The overloading effects are rationalized by a higher local viscosity close to the membrane and we suggested an analytical solution to explain the decrease of the zone velocity due to the viscosity effect. These findings could be relevant in practice as they demonstrate that when high sample load is necessary (e.g., when AF4 is used as a semi-preparative method or to improve detection), the loading capacity could be considerably increased with changes in temperature or in the solvent (e.g., ionic strength, additives) that aim to decrease the dependency of the viscosity on the sample concentration.

## Electronic supplementary material

ESM 1 (PDF 204 kb)
