# Supplementary material for: Recovery, overloading, and protein interactions in asymmetrical flow field-flow fractionation
Source: Anal Bioanal Chem. 2019 Feb 21;411(11):2327–38. doi: 10.1007/s00216-019-01673-w (PMC6459789; doi:10.1007/s00216-019-01673-w)
Supplement: Supplementary file 1 — (PDF 204 kb) [file 216_2019_1673_MOESM1_ESM.pdf]

Analytical and Bioanalytical Chemistry

Electronic Supplementary Material

Recovery, overloading and protein interactions in asymmetrical flow field-flow fractionation

Maria Marioli, Wim Th. Kok

An estimation can be derived for the effect of the viscosity increase, caused by high concentrations of the proteins close to the accumulation wall, on the velocity of the protein zone. First, it is assumed that the concentration of the protein as a function of the distance from the wall x can be described in the usual way:

$$c(x) = c_0 \cdot \exp(-\frac{x}{\ell}) \qquad \text{S1}$$

where c_0 is the protein concentration at the wall and ℓ the average layer thickness (see Eq. (2)). The dependency of the diffusion coefficient on the concentration of the protein, as has been found in experimental studies [32] is not taken into account here.

Further it is assumed that the shear in the protein layer is constant and equal to the shear in the linear part of the parabolic profile of the unperturbed channel flow, τ_0. The unperturbed shear in a thin channel close to the wall can be found as:

$$\tau_0 = \frac{6\langle v \rangle}{w}\eta_0 \qquad \text{S2}$$

where $\langle v \rangle$ is the average channel flow velocity, w the height of the channel and η_0 the viscosity of the carrier solution. When the viscosity is a function of the protein concentration, the local flow velocity close to the wall $v(x)$ can be evaluated from:

$$\frac{dv(x)}{dx} = \frac{\tau_0}{\eta(c)} \qquad \text{S3}$$

where x is the distance from the membrane and $\eta(c)$ the concentration-dependent viscosity of the carrier solution. By substitution of Eqs. (S1) and (4) in Eq. (S3), and integration over x, the velocity profile can be found. The result of the integration is:

$$v(x) = \frac{\tau_0 \ell}{\eta_0} \left\{ \frac{1}{2} ln \frac{1 + k_1 c_0 \exp\left(-\frac{x}{\ell}\right) + k_2 c_0^2 \exp\left(-\frac{2x}{\ell}\right)}{\exp\left(-\frac{2x}{\ell}\right)(1 + k_1 c_0 + k_2 c_0^2)} - \frac{k_1}{\sqrt{4k_2 - k_1^2}} arctan \frac{c_0(1 - \exp\left(-\frac{x}{\ell}\right))\sqrt{4k_2 - k_1^2}}{2 + k_1 c_0(1 + \exp\left(-\frac{x}{\ell}\right)) + 2k_2 c_0^2 \exp\left(-\frac{x}{\ell}\right)} \right\} \qquad \text{S4a}$$

when $4k_2 - k_1^2 > 0$, and

$$v(x) = \frac{\tau_0 \ell}{\eta_0} \left\{ \frac{1}{2} ln \frac{1 + k_1 c_0 \exp\left(-\frac{x}{\ell}\right) + k_2 c_0^2 \exp\left(-\frac{2x}{\ell}\right)}{\exp\left(-\frac{2x}{\ell}\right)(1 + k_1 c_0 + k_2 c_0^2)} \right.$$

$$\left. - \frac{k_1}{\sqrt{-4k_2 + k_1^2}} arctanh \frac{c_0(1 - \exp\left(-\frac{x}{\ell}\right))\sqrt{-4k_2 + k_1^2}}{2 + k_1 c_0 (1 + \exp\left(-\frac{x}{\ell}\right)) + 2k_2 c_0^2 \exp\left(-\frac{x}{\ell}\right)} \right\} \qquad \text{S4b}$$

when $4k_2 - k_1^2 < 0$.

The zone velocity (the average velocity of the protein molecules) can then be found by integration of the product of the concentration and the velocity profiles:

$$v_z = \int_0^\infty v(x) \cdot c(x) dx \, / \int_0^\infty c(x) dx \qquad \text{S5}$$

The result of the integration is:

$$v_z = v_{z,0} \frac{2}{c_0 \cdot \sqrt{4k_2 - k_1^2}} \cdot arctan\left(\frac{c_0 \cdot \sqrt{4k_2 - k_1^2}}{2 + k_1 c_0} \right) \qquad \text{S6a}$$

when $4k_2 - k_1^2 > 0$, and

$$v_z = v_{z,0} \frac{2}{c_0 \cdot \sqrt{-4k_2 + k_1^2}} \cdot arctanh\left(\frac{c_0 \cdot \sqrt{-4k_2 + k_1^2}}{2 + k_1 c_0} \right) \qquad \text{S6b}$$

when $4k_2 - k_1^2 < 0$.

In equation (S6) $v_{z,0}$ is the velocity of the zone under ideal conditions ($c_0 \rightarrow 0$). When only the linear effect of the concentration on the viscosity (k_1) is taken into account, the equation for the velocity profile simplifies to:

$$v(x) = \frac{\tau_0 \ell}{\eta_0} \cdot ln \frac{1 + k_1 c_0 \cdot \exp(-\frac{x}{\ell})}{\exp\left(-\frac{x}{\ell}\right) + k_1 c_0 \cdot \exp(-\frac{x}{\ell})} \qquad \text{S7}$$

and for the zone velocity the result is:

$$v_z = v_{z,0} \cdot \frac{\ln(1 + k_1 c_0)}{k_1 c_0} \qquad \text{S8}$$

In Fig. S1 the predicted effect of the sample load on the zone velocity under overload conditions is shown for two hypothetical cases. When the increase of the viscosity with the protein concentration is linear (when the contribution of the single molecule to the viscosity is higher than of the pairwise interactions), a gradual decrease of the zone velocity with the mass

load is expected. However, when the increase is more in a quadratic way (pairwise interactions are the most important contribution to the viscosity) there is a more sudden change of the velocity above a certain threshold mass load.

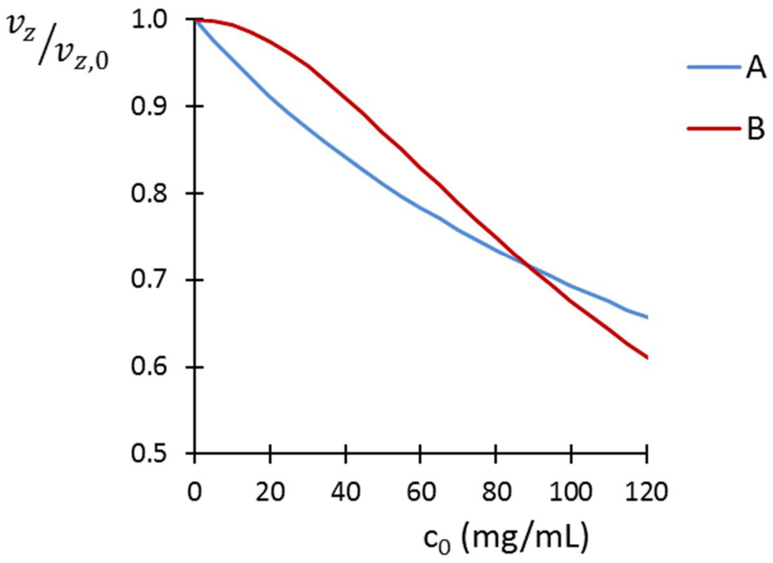

Fig. S1 Zone velocity as a function of the protein concentration at the accumulation wall

A: k_1 = 0.01 mL/mg, k_2 = 0; B: k_1 = 0, k_2 = 0.0002 mL^2 / mg^2
